# *L1R, A27L, A33R* and *B5R* vaccinia virus genes expressed by fowlpox recombinants as putative novel orthopoxvirus vaccines

**DOI:** 10.1186/1479-5876-11-95

**Published:** 2013-04-11

**Authors:** Sole Maria Pacchioni, Massimiliano Bissa, Carlo Zanotto, Carlo De Giuli Morghen, Elena Illiano, Antonia Radaelli

**Affiliations:** 1 Department of Medical Biotechnologies and Translational Medicine, University of Milan, via Vanvitelli 32, 20129 Milan, Italy; 2 Cellular and Molecular Pharmacology Section, CNR Institute of Neurosciences, University of Milan, via Vanvitelli 32, 20129 Milan, Italy; 3 Department of Medical Biotechnologies and Translational Medicine, University of Milan, via Vanvitelli, 32, 20129 Milan, Italy; 4 Laboratory of Molecular Virology and Recombinant Vaccine Development, Department of Medical Biotechnologies and Translational Medicine, University of Milan, Via Vanvitelli 32, 20129 Milan, Italy

**Keywords:** Smallpox vaccine, Orthopoxvirus infections, Fowlpox recombinants, Transgene expression

## Abstract

**Background:**

The traditional smallpox vaccine, administered by scarification, was discontinued in the general population from 1980, because of the absence of new smallpox cases. However, the development of an effective prophylactic vaccine against smallpox is still necessary, to protect from the threat of deliberate release of the variola virus for bioterrorism and from new zoonotic infections, and to improve the safety of the traditional vaccine. Preventive vaccination still remains the most effective control and new vectors have been developed to generate recombinant vaccines against smallpox that induce the same immunogenicity as the traditional one. As protective antibodies are mainly directed against the surface proteins of the two infectious forms of vaccinia, the intracellular mature virions and the extracellular virions, combined proteins from these viral forms can be used to better elicit a complete and protective immunity.

**Methods:**

Four novel viral recombinants were constructed based on the fowlpox genetic background, which independently express the vaccinia virus L1 and A27 proteins present on the mature virions, and the A33 and B5 proteins present on the extracellular virions. The correct expression of the transgenes was determined by RT-PCR, Western blotting, and immunofluorescence.

**Results and conclusions:**

Using immunoprecipitation and Western blotting, the ability of the proteins expressed by the four novel FP_*L1R*_, FP_*A27L*_, FP_*A33R*_ and FP_*B5R*_ recombinants to be recognized by VV-specific hyperimmune mouse sera was demonstrated. By neutralisation assays, recombinant virus particles released by infected chick embryo fibroblasts were shown not be recognised by hyperimmune sera. This thus demonstrates that the *L1R, A27L, A33R* and *B5R* gene products are not inserted into the new viral progeny. Fowlpox virus replicates only in avian species, but it is permissive for entry and transgene expression in mammalian cells, while being immunologically non–cross-reactive with vaccinia virus. These recombinants might therefore represent safer and more promising immunogens that can circumvent neutralisation by vector-generated immunity in smallpox-vaccine-experienced humans.

## Background

Preventive vaccination still remains the most effective control against orthopoxvirus (OPXV) infections, as it can elicit neutralising antibodies against an incoming virus. The traditional smallpox vaccine was administered by scarification, but its use was discontinued in the general population from 1980, because of the absence of new smallpox cases. However, attenuated strains of vaccinia virus (VV) have been produced and tested in humans in attempts to develop strains with lower reactogenicity and fewer side effects. Indeed, in spite of the discontinuation of the smallpox vaccination programmes, the threat of deliberate release of variola virus for bioterrorism and the need for protection from new zoonotic infections still remain [1-4]. Also, the monkeypox virus (MPXV) resembles smallpox in the severity of its symptoms, and it might be a potential bioweapon if cowpox and MPXV can adapt to grow and spread in humans [5].

VV recombinants that express different transgenes have already been used in clinical studies for the prevention and immunotherapy of different infectious diseases. However, although extremely effective, VV raises safety concerns due to its high reactogenicity, its ability to spread to non-vaccinated subjects, and its moderate to severe side effects [6], especially in immune-compromised individuals [7]. Such side effects are not acceptable in a post-endemic smallpox era [6,8]. Clinical trials have also been performed with the attenuated VV-derived Lister clone LC16m8 and the modified vaccinia Ankara (MVA). LC16m8, which replicates in humans, showed protective efficacy in animal models, and was safely used for over 50,000 children in Japan in 1974 [9]. MVA has an extensive history of safety in humans, it is immunogenic and efficacious in both mice and non-human primates, where it can also protect against MPXV, and it is a leading candidate for an alternative smallpox vaccine [10-13]. However, as MVA replication in mammals is only partially abortive [14], and induces lower immunogenicity than the traditional smallpox vaccine [15], the search for new alternatives is still ongoing [16,17]. Failure of protection with MVA has been demonstrated in animals with CD4/CD8 combined immunodeficiency [6] and in Rhesus macaques infected with simian immunodeficiency virus with a very low cell count of the immune repertoire [18].

The VV antigens that protect against smallpox are not completely known. However, analysis of the immune responses after immunisation with traditional vaccines has shown that the neutralising antibodies are mainly directed against the surface proteins of the two infectious forms of OPXVs: the intracellular mature virions (MVs) and the extracellular virions (EVs). MVs are released after cell lysis, and they are responsible for the host-to-host spread because of their stability in the extracellular environment. In contrast, EVs are wrapped by an additional envelope, and they have an important role in cell-to-cell spread; i.e., in the dissemination within the host [19,20]. In particular, the L1 and A27 proteins on MVs and the A33 and B5 proteins on EVs are involved in the attachment, fusion and penetration of the virus into target cells [20,21]. Combined DNA-based vaccines expressing these four proteins are more protective than vaccines carrying individual immunogens [22,23], possibly because of the induction of synergistic antibodies that can act at the different infection phases, both at the initial exposure, and during viral dissemination [24,25].

Although other immunogens have been used to induce protective immunity, when these four genes have been delivered as DNA expression plasmids [22] or as virus-like replicons [17], they have already been shown to induce functional antibodies and to protect mice against VV parenteral/ intranasal challenge, and monkeys against MPXV intravenous challenge [26,27].

In the present study, four novel recombinants were constructed based on the fowlpox (FP) genetic background that express the VV *L1R, A27L, A33R* and *B5R* genes independently. Their correct expression was determined by RT-PCR, Western blotting and immunofluorescence, both in replication-permissive chick embryo fibroblasts (CEFs) and in non-permissive mammalian Vero and MRC-5 cells. The ability of the proteins expressed by the four novel recombinants to be recognized by VV-specific hyperimmune mouse sera was demonstrated by immunoprecipitation followed by Western blotting (IP/WB) using hyperimmune mouse serum. The recognition by VV-specific antibodies of a mixture of the four FP recombinants (4FPmix) was tested using a plaque-reduction neutralisation assay.

FP vectors are replication-restricted to avian species [28], but they are permissive for entry and transgene expression in mammalian cells, while being immunologically non–cross-reactive with VV. They therefore represent safer immunogens [29], as they can circumvent neutralisation by vector-generated immunity in smallpox-vaccine-experienced humans. The advanced replication cycle, long transgene expression, and balanced Th1/Th2 cytokine induction of FP-based recombinants [28] might elicit a more effective immune response.

## Methods

### Cells

Specific-pathogen-free primary CEFs were grown in Dulbecco’s modified Eagle’s medium (DMEM) supplemented with 5% heat-inactivated calf serum (Gibco Life Technologies, Grand Island, NY, USA), 5% Tryptose Phosphate Broth (Difco Laboratories, Detroit, MI, USA), 100 U/ml penicillin and 100 mg/ml streptomycin. Green monkey kidney (Vero) cells, and normal human lung fibroblasts (MRC-5 cells) were grown in DMEM supplemented with 10% heat-inactivated calf serum, 100 U/ml penicillin and 100 mg/ml streptomycin.

### Construction of the recombinants

Four FP-based recombinants expressing the VV MV L1 and A27 proteins and the EV A33 and B5 proteins were obtained by *in-vitro* homologous recombination [30-32], with minor modifications. FPwt was obtained by J. Taylor (Wadsworth Center, NY State Dept. of Health, Albany, NY) [29]. Molecular cloning of pFP_*A33R*_ and preparation of the FP_*A33R*_ recombinant have already been described [33]. For FP_*L1R*,_ FP_*A27L*_ and FP_*B5R*_, these genes were PCR-amplified from the Lancy strain of VV DNA (Berna Biotech, courtesy of M. R. Capobianchi, L. Spallanzani National Institute for Infectious Diseases, Rome, Italy). They were separately inserted into the pcDNA3 and pBSII plasmids (*L1R*), the pBSII plasmid (*A27L*), or the pcDNA3 and pAFTd plasmids (*B5R*). Insertion into the pFP_MCS_ recombinant plasmid was performed downstream of the VV H6 early/ late promoter [34], inside the 3-β-hydroxysteroid dehydrogenase 5-delta 4 isomerase gene interrupted by a multiple cloning site. The amplification of *L1R* was carried out using the forward V182 (5’ GGG AAG CTT TTA AAT GGG TGC CGC AGC AAG CAT ACA 3’) and reverse V183 (5’ GGG CTC GAG ATT TTC AGT TTT GCA TAT CCG TGG TAG 3’) primers. For *A27L*, the forward V354 (5’ CCC GGG AAG CTT AAT GGA CGG AAC TCT TTT C 3’) and reverse V353 (5’ TTT TGG TAC CAT AAA AAT TAC TCA TAT GGG CGC CG 3’) primers were used. For *B5R*, the forward V184 (5’ GGG AAG CTT AAA AAT GAA AAC GAT TTC CGT TGT TAC 3’) and reverse V185 (5’ GGG CTC GAG ATA TTT ACG GTA GCA ATT TAT GGA ACT 3’) primers were used. Amplifications were performed as described previously [35], using 2.5 mM MgCl_2_ and 2 mM MgSO_4_, with annealing at 61°C for 30 s (*L1R* and *A27L*), or at 57°C for 30 s (*B5R*) and extension at 72°C for 45 s. The β-actin reference gene was amplified using the forward V84 (5’ CTG ACT ACC TCA TGA AGA TCC T 3’) and reverse V85 (5’ GCT GAT CCA CAT CTG CTG GAA 3’) primers, with 1 mM MgSO_4_ and with annealing at 60°C for 30 s. The plasmid DNAs were purified and the genes were sequenced (Genenco, MMedical, Milan, Italy), to exclude any mutations arising from the PCR amplification. The plasmids are designated as pFP_*L1R*_ (8,992 bp), pFP_*A27L*_ (8,568 bp), pFP_*A33R*_ (8,764 bp), and pFP_*B5R*_ (9,173 bp). Recombinants were obtained by *in-vitro* homologous recombination in CEFs, using FPwt and the different pFP recombinants, with minor modifications. Recombinant plaques were identified by autoradiography after hybridisation with [^32^P]-labelled specific probes, and then subjected to multiple cycles of plaque purification. One clone was selected for correct and high expression of each gene by Western blotting, using specific antibodies. The recombinant viruses were amplified in CEFs, purified on discontinuous sucrose density gradients, and titrated essentially as already described [36]. Briefly, the cells were harvested, ultracentrifuged at 30,000× *g* for 2 h at 4°C, and the pellets resuspended in 1 mM Tris, 150 mM NaCl, 1 mM EDTA, pH 7.4. The pellet then had 0.06% trypsin added, and was incubated for 5 min at 37°C, and the virus was released from the cells by sonication. The supernatant was overlaid onto a discontinuous 30% to 45% (w/w) sucrose gradient, in the same buffer. After ultracentrifugation at 38,000× *g* for 1 h, the viral band at the interface was recovered, diluted with 1 mM Tris-HCl, pH 9, and pelletted at 67,000× *g* for 1 h. The purified virus was resuspended in Ca^++^- and Mg^++^-free phosphate-buffered saline (PBS^-^), disaggregated by sonication, aliquoted, and frozen at -80°C until use.

### RT-PCR

The expression of *L1R, A27L, A33R* and *B5R* was investigated by RT-PCR. The cells were infected with 1 plaque-forming unit (PFU)/cell of the FP_*L1R*_, FP_*A27L*_, FP_*A33R*_ or FP_*B5R*_ recombinants, and the mRNAs were extracted 24 h post-infection (p.i.) from CEFs and Vero and MRC-5 cells, as described previously [28]. Briefly, 50 ng RNA from each sample was used in a final volume of 10 μl, in the presence of 1 μM of each primer, 200 mM of each dNTP, 0.1 U/μl *Thermus flavus* DNA polymerase, and 0.1 U/μl avian myeloblastosis virus reverse transcriptase, using the V182/V183 primers for *L1R* (779 bp), V354/V353 for *A27L* (363 bp), V186/V187 for *A33R* (584 bp), V184/V185 for *B5R* (980 bp), and V84/85 (518 bp) for β-actin detection under the conditions described above. RNA from FPwt-infected cells was used as a negative control. The RT-PCR products were quantified using the ImageJ software [37].

### Western blotting

To determine whether the L1, A27, A33, and B5 proteins were expressed by the recombinants at the same levels in the different cell lines, CEFs and Vero and MRC-5 cells were infected (10 PFU/cell) and examined by Western blotting, as already described [38]. The blotted nitrocellulose membranes were incubated overnight at 4°C with 1:100 dilutions of the primary antibody. Alternatively, two different specific antibodies (*Bei*resources, Manassas, VA, USA) were used for each gene: a mouse monoclonal antibody followed by goat anti-mouse horseradish-peroxidase-conjugated serum, and a rabbit polyclonal antibody followed by goat anti-rabbit horseradish-peroxidase-conjugated serum (1:2,000 dilution; DakoCytomation, Carpinteria, CA, USA). After a 1-h incubation and 2 h of washes, the proteins were revealed using the ECL system (EuroClone, Pero, Milan, Italy). Cells infected with FPwt were used as the negative control.

### Immunoprecipitation/ Western blotting analysis

Conventional Western blotting was also performed after immunoprecipitation of cell lysates using specific antibodies (IP/WB). Vero cells were infected as for Western blotting, and immunoprecipitation was performed essentially as already described [35], with minor modifications. Sixteen hours p.i., the cells were harvested by resuspension in 1 ml lysis buffer (150 mM NaCl, 1 mM EDTA, 10 mM Tris-HCl, pH 7.4, 0.2 mg/ml PMSF, 1% NP40, 0.01% sodium azide) per Petri dish, and 0.6 TIU aprotinin (Sigma, St Louis, MO, USA). The lysate was clarified by centrifugation at 9,000× *g* for 20 min at 4°C, and immunoprecipitation was performed with 10 μl anti-IHD-J mouse hyperimmune serum, from our laboratory. The proteins were resolved using 15% SDS-PAGE, identified using a polyclonal antibody (*Bei*resources), and revealed using the ECL system.

### Immunofluorescence

Protein expression by the recombinant viruses was also examined by immunofluorescence, which was carried out in CEFs and Vero and MRC-5 cells, essentially as described previously [35]. In particular, after infection at 37°C for 1 h with 1 or 3 PFU/cell (depending on the different cytopathogenicity of the recombinants), the cells were grown for 6 or 15-18 h before immunofluorescence. The cells were fixed either with only fresh 2% paraformaldehyde in PBS^-^ for 10 min, for membrane immunofluorescence, or with paraformaldehyde followed by 100% cold acetone for 5 min at -20°C, for cytoplasmic immunofluorescence. The samples were incubated for 1 h with monoclonal or polyclonal antibodies (*Bei*resources) (Table 1). FITC 1:100-diluted anti-mouse or anti-rabbit secondary antibodies (Cappel, MP Biomedicals, Inc., Aurora, OH, USA) were used. Cells infected with FPwt were the negative controls. The samples were viewed under a Zeiss Axioskop epifluorescence microscope.

**Table 1:** Antibody dilutions for IF

**FP recombinant**	**MoAb**	**PolyAb**
**FP**_***L1R***_	neg	1:50
**FP**_***A27L***_	neg	1:50
**FP**_***A33R***_	1:50	1:50
**FP**_***B5R***_	1:20	1:200

MoAb, mouse monoclonal antibody.

PolyAb, rabbit polyclonal antibody.

neg, no positive IF at any dilution.

### IHD-J and preparation of the four FP recombinants mixture

The IHD-J strain of VV was kindly obtained from S. Dales (University of Western Ontario, London, Canada) [39] grown in Vero cells, and used to repeatedly infect Balb/c mice (1 × 10^5^ PFU/mouse) via the airways, to obtain VV-specific hyperimmune serum. A mixture of the four FP recombinants (4FPmix) was also prepared in CEFs, by co-infection with the four FP viruses (3 PFU/cell/each recombinant virus). The IHD-J and the 4FPmix viruses were amplified in Vero cells and CEFs, respectively, purified on discontinuous sucrose density gradients, and titrated as already described.

### Virus neutralisation assays

Recognition of the 4FPmix by VV-specific mouse antibodies was tested using the virus neutralisation assay. VV IHD-J hyperimmune mouse serum was used to determine the inhibition of the infectivity of the 4FPmix. Neutralisation assays were performed by pre-incubating the 4FPmix with an equal volume of heat-inactivated hyperimmune serum, for 1 h at 37°C. Pre-immune serum was used as a negative control. The IHD-J virus was used in a parallel test with both pre-immune and hyperimmune serum. The sera were diluted starting from a 1:20 dilution in DMEM. The viral inoculum was adjusted to give approximately 10^2^ PFU/Petri dish. Infection was allowed to proceed for 1 h at 37°C. The cells then had 5 ml DMEM with agarose LE 0.7% (SeaKem, FMC BioProducts, Rockland, ME, USA) added. The plaque numbers were counted on day 4 p.i., after adding an agarose layer containing 1.5% neutral red (Gibco). The neutralisation is expressed as the percentage of reduction of plaque numbers *versus* the control, where the viral inoculum was incubated with no serum.

## Results

### Transcript expression by the FP recombinants is similar in the different cell lines

**Figure 1:**
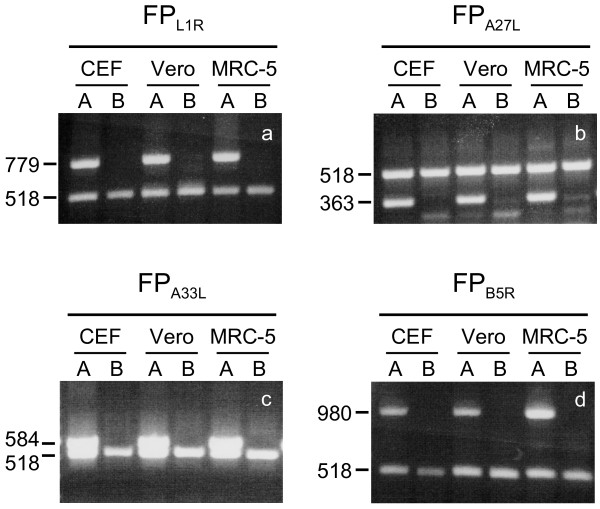
**Expression of the FP**_***L1R***_**, FP**_***A27L***_**, FP**_***A33R***_**and FP**_***B5R***_**transcripts by RT-PCR in replication-permissive and non-permissive cells.** Following infection of CEFs and Vero and MRC-5 cells with the four recombinants, transcript expression was detected after overnight infection, as 779-bp (panel **a**), 363-bp (panel **b**), 584-bp (panel **c**) and 980-bp (panel **d**) fragments. Expression of human β-actin is shown as a 518-bp band (panels **a**-**d**). No specific band is seen for cells infected with FPwt, which was used as a negative control (lanes B).

After RNA isolation from the infected CEFs and Vero and MRC-5 cells, the transcripts were detected by RT-PCR after an overnight incubation, as 779-bp, 363-bp, 584-bp and 980-bp fragments in all of the cell lines infected by the FP_*L1R*_, FP_*A27L*_, FP_*A33R*_ and FP_*B5R*_ recombinants, respectively (Figure 1, lanes A). As determined by densitometric analysis, similar levels of expression were observed in the different cells for each recombinant, except for FP_*B5R*_, that expressed the *B5R* mRNA 2.1 times more in human MRC-5 cells than in CEF and Vero cells. The amplification of human β-actin RNA is also shown as a 518-bp band (Figure 1, lanes A). As expected, the FPwt-infected cells used as a negative control did not show any specific band (Figure 1, lanes B).

### The recombinant proteins are expressed by all of the FP recombinants in all of the different cell lines, although at different levels

**Figure 2:**
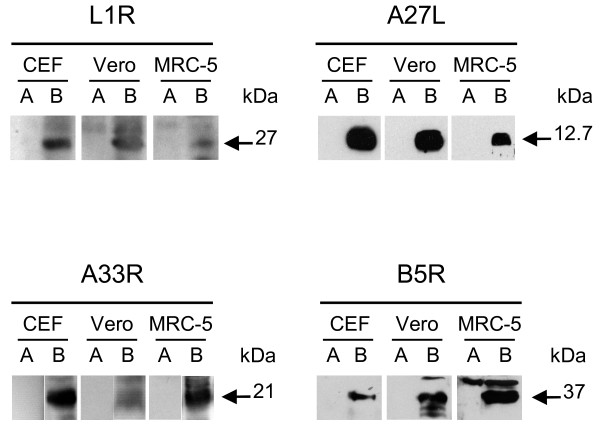
**Transcript expression by Western blotting in replication-permissive and non-permissive cell lines.** All of the different cell lines, the CEFs and Vero and MRC-5 cells, were infected with 10 PFU/cell of FP_*L1R*_, FP_*A27L*_, FP_*A33R*_ or FP_*B5R*_, and harvested 15-18 h p.i.. Rabbit polyclonal primary antibodies recognised the corresponding proteins, in all of the cell lines (lanes B). The expression levels were quantified by densitometric determinations. No specific bands were seen when the cells were infected with FPwt (lanes A).

The FP recombinants expression was verified by Western blotting on lysates of both replication-permissive CEFs and non-permissive Vero and MRC-5 cells, which were infected separately with FP_*L1R*_, FP_*A27L*_, FP_*A33R*_ and FP_*B5R*_, or with FPwt. The polyclonal primary antibodies recognised specific 27-kDa, 12.7-kDa, 21-kDa and 37-kDa bands, which corresponded to the L1, A27, A33 and B5 proteins, respectively, in the infected CEFs and Vero and MRC-5 cells (Figure 2, lanes B). As determined by densitometric analysis, the expression levels for FP_*L1R*_ were 4.2-fold and 3.9-fold higher in CEFs and Vero cells than in MRC-5 cells, those for FP_*A27L*_ were 2.9-fold and 2.5-fold higher in CEFs and Vero cells than in MRC-5 cells, those for FP_*A33R*_ were 2.6-fold and 3-fold higher in CEFs and MRC-5 cells than in Vero cells, and those for FP_*B5R*_ were 2.1-fold and 3.1-fold higher in Vero and MRC-5 cells than in CEFs (Figure 2; lanes B). No specific bands were recognised when the cells were infected with FPwt (Figure 2; lanes A). The corresponding monoclonal antibodies showed similar specificity, but lower binding activity (data not shown).

### Vaccinia hyperimmune mouse serum recognises FP-expressed heterologous proteins

**Figure 3:**
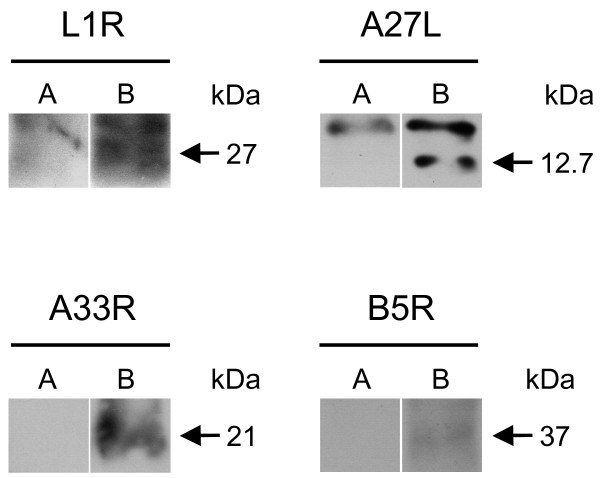
**Immunoprecipitation/Western blotting.** To verify the recognition of the FP-expressed proteins also by VV-specific hyperimmune mouse sera, immunoprecipitation was performed, followed by Western blotting. After infection with FP_*L1R*_, FP_*A27L*_, FP_*A33R*_ or FP_*B5R*_, the specific proteins were immunoprecipitated by the hyperimmune sera (lanes B). No specific bands were seen in cells infected with FPwt (lanes A).

IP/WB was used to test whether the hyperimmune mouse sera could also recognise the FP-expressed proteins. After infection of the Vero cells with the FP_*L1R*_, FP_*A27L*_, FP_*A33R*_ and FP_*B5R*_ recombinants, the specific proteins were immunoprecipitated from cell lysates using sera from mice that had been repeatedly immunised with VV IDH-J (Figure 3, lanes B). No specific bands were seen for cells infected with FPwt (Figure 3, lanes A).

### Different localisation of the foreign proteins is detected by immunofluorescence

**Figure 4:**
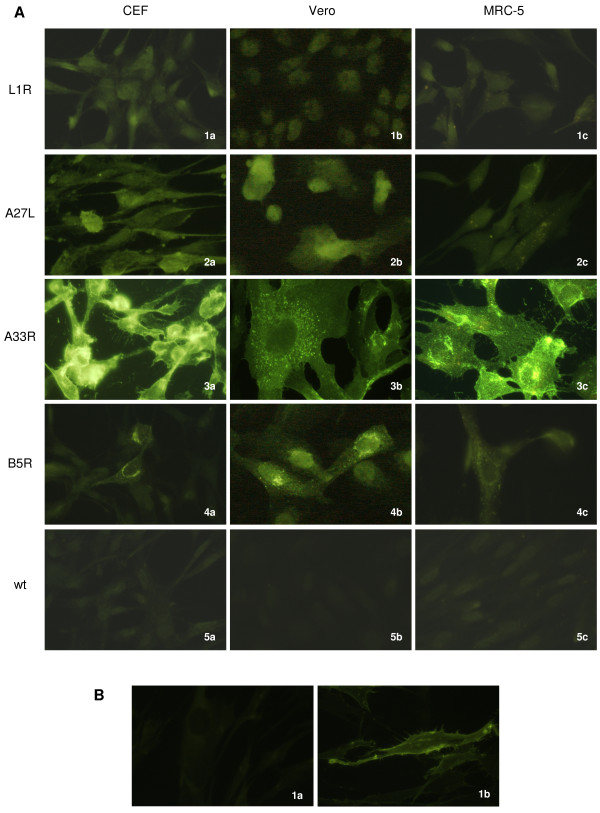
**Heterologous protein localisation by immunofluorescence.** (**A**) Intracellular immunofluorescence was detected in all of the cell lines, with differences between the proteins ascribed to MV or EV particles. In cells infected with FP_*L1R*_ or FP_*A27L*_ the cytoplasmic immunofluorescence was diffuse (panels 1**a**-**c**, 2**a**-**c**), and in FP_*L1R*_-infected cells, the difference with the negative control was very low. Conversely, specific, granular perinuclear localisation was shown by the cells infected with FP_*A33R*_ or FP_*B5R*_ (panels 3**a**-**c**, 4**a**-**c**), which was particularly evident in MRC-5 cells infected with FP_*A33R*_. FPwt-infected cells were negative (panels 5**a**-**c**). (**B**) MRC-5 cells showed membrane immunofluorescence only after infection with the FP_*A33R*_ recombinant (panel 1**b***vs.* 1**a**).

To identify differences in the heterologous protein expression and subcellular localisation in the different cell lines, immunofluorescence was performed at 15-18 h p.i. in CEFs and Vero and MRC-5 cells infected with the FP_*L1R*_, FP_*A27L*_, FP_*A33R*_ and FP_*B5R*_ recombinants, or with FPwt (Figure 4). The cells infected with FP_*L1R*_ and FP_*A27L*_ showed diffuse cytoplasmic fluorescence (Figure 4A, 1a-c, 2a-c), although the specific staining with FP_*L1R*_ was only slightly greater than the negative control, in all of the cell lines. Conversely, there was specific, greater, granular perinuclear and cytoplasmic localisation in the cells infected with FP_*A33R*_ and FP_*B5R*_ (Figure 4A, 3a-c, 4a-c), which was particularly evident in the MRC-5 cells infected with FP_*A33R*_. FPwt-infected cells were always negative (Figure 4A, 5a-c). Immunofluorescence performed at 6 h p.i. gave similar results (data not shown). Membrane immunofluorescence was present only after infection with the FP_*A33R*_ recombinant in MRC-5 cells (Figure 4B, 1b *vs.* 1a). Polyclonal and monoclonal antibodies were used at different dilutions for the different recombinants (Table 1).

### The 4FPmix is not neutralised by VV hyperimmune serum

**Figure 5:**
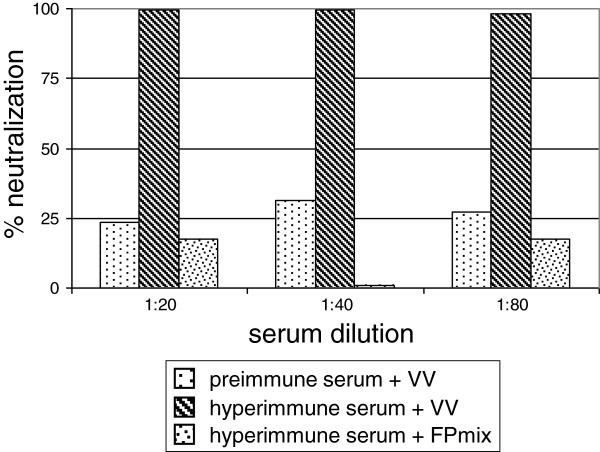
**Virus neutralisation assay.** A mixture of all of the four FP recombinants (4FPmix) was incubated with IHD-J hyperimmune serum to determine whether the proteins expressed by the recombinants were localised on the surface of the new viral progeny. High neutralising activity was detected only in the samples where IHD-J was pre-incubated with IHD-J-hyperimmune mouse serum.

To determine whether the FP recombinant virions produced by the CEFs were carrying the structural products of the four transgenes on their surface, the 4FPmix, containing a mixture of all of the FP recombinants, was incubated with VV IHD-J hyperimmune serum. No reduction in plaque numbers was detected, as compared to samples where pre-immune serum was incubated with the IHD-J virus. High neutralising activity was seen in samples where IHD-J was pre-incubated with IHD-J hyperimmune serum (Figure 5).

## Discussion

Development of alternative replication-deficient vaccine candidates against smallpox and other zoonotic OPXV infections is still needed as a defence against new poxvirus outbreaks as well as to reduce the adverse reactions of traditional vaccines and the limited immune responses in VV-experienced individuals. Along with discontinuation of the smallpox vaccination campaign and the decline in herd immunity in most countries, an increase in MPXV infections has been described [40,41], which has mostly occurred in children, young adults, and immune-compromised patients [42]. MPXV is widely distributed in a variety of African rodents, and particularly in squirrels, which might be the reservoir [43], and it has the potential to be used as a bio-weapon [41,44,45]. Outbreaks of human MPXV infections were also reported in the Unites States in 2003, with 69 people infected following the importation of MPXV-infected rodents from West Africa. Also, in the Republic of Congo in 1996-1997, there were 92 cases and 3 deaths [46], which might have been related to the overall vanishing immunity against poxviruses throughout the world community.

The available smallpox vaccines based on VV show relatively high rates of adverse side effects, and new strategies should therefore be devised to improve the safety of traditional smallpox vaccines. Replication-deficient vaccines should reduce the risks for animal handlers that arise from accidental exposure to cowpoxvirus and other OPXV, limit the OPXV-infection of domestic animals, and should be useful for protective immunization in the event of intentional or accidental release of variola virus.

In the present study, we have described the characterisation of four new FP recombinants, FP_*L1R*_, FP_*A27L*_, FP_*A33R*_ and FP_*B5R*_, that express the VV *L1R, A27L, A33R* and *B5R* genes. We demonstrated that: (i) all of these recombinants can express the proteins correctly, although at different levels in different cell lines, as revealed by RT-PCR and Western blotting; (ii) MV and EV proteins show different subcellular localisations; (iii) recombinant FP virions (4FPmix) are resistant to neutralisation by VV-specific serum; and (iv) the four FP recombinants express functional foreign proteins that are immunoprecipitated by hyperimmune serum from IHD-J-immunised mice.

Using Western blotting, heterologous VV-specific proteins were detected in cells infected by the four recombinants. Their expression in human and non-human primate cells was often higher than in CEFs, where the virus replicates. This can be ascribed to the cytocidal effect of the FP recombinants in avian cells, although it is not clear why this occurs only for the expression of specific genes. In particular, the higher transgene expression by FP_*B5R*_ in human MRC-5 cells confirms the results already shown by RT-PCR. IP/WB was used to overcome the difficulty of revealing cell-expressed antigens by conventional Western blotting, especially when using mouse or human hyperimmune sera. This assay, which better avoids nonspecific binding to nitrocellulose and makes the native proteins detectable, showed that these proteins can be recognised by VV-specific hyperimmune mouse serum.

By immunofluorescence, the heterologous proteins were mainly localised at the cytoplasmic level for all of the recombinants, and also at the membrane surface when MRC-5 cells were infected with FP_*A33R*_. Although no specific differences were observed among the cells lines, the expression of the proteins belonging to MV particles was always lower than the expression of the proteins belonging to EV particles. In particular, the intracellular expression of A33 and B5 was granular, perinuclear and cytoplasmic, which confirms previously reported data, and demonstrates their high expression in the juxtanuclear area and Golgi region [47]. Membrane fluorescence was only detectable after infection with FP_*A33R*_. This also confirms previous data showing the spontaneous translocation of the A33 protein to the plasma membrane, which, using a FP_*B5R*_ recombinant, was only observed in very low amounts by immunoelectron microscopy, but not by immunfluorescence [47]. The very low fluorescence detected after FP_*L1R*_ infection can either be ascribed to limited protein expression, which was not confirmed by the Western blotting, or to the low avidity of the antibody. This is also confirmed by other studies, where low expression was overcome by the use of the phorbol ester 12-O-tetradecanoylphorbol-13-acetate (TPA) [48].

The absence of neutralising activity of IHD-J-specific hyperimmune mice sera against the virus particles released by CEFs infected with the four FP recombinants suggests that the L1, A27, A33 and B5 gene products may not be inserted into the envelope of the new viral progeny. This enhances the importance of these FP recombinants for their use in individuals who have already been vaccinated against smallpox, where these can be administered as a recall. As human peripheral blood monocytic cells and macrophages are permissive for penetration of the recombinants and expression of foreign proteins [28], antigen cross-presentation should occur, which should result in complete humoral and cellular immune responses.

## Conclusions

To avoid lytic infection, ulceration and scab formation after dermal scarification with VV for smallpox prophylaxis, FP recombinants represent alternative safer immunogens. This arises from their natural host-range restricted replication to avian species [49], their correct transgene expression in mammalian cells, and their ability to elicit a complete immune response in vaccinated hosts [50]. Although these *L1R, A27L, A33R* and *B5R* VV-derived genes have already been successfully expressed using recombinant DNA [22,48], the use of avipox recombinants expressing these genes might provide better control against zoonotic OPXV infections, will offer advances in the fight against the threat of bioterrorism, and can also be used in VV-experienced individuals.

## Competing interests

The authors declare that they have no competing interests.

## Authors’ contributions

SP performed the production and purification of the FP recombinants, the Western blotting, and the IP/WB. MB performed the production and purification of the FP recombinants, and the immunofluorescence assays. CZ performed the molecular cloning, prepared the primary cell cultures, prepared the Figures, and analysed the data and the study results. CDGM conceptualised and supervised the whole study. EI performed the RT-PCR and animal immunisations. AR designed the study, performed the neutralisation assays, assisted in the animal immunisations, analysed the data, interpreted the study results, and prepared the manuscript. All of the authors have read and approved the present version of the manuscript.

## References

[B1] Whitley RJ Smallpox: a potential agent of bioterrorism Antiviral Res 2003 57 7 12 10.1016/S0166-3542(02)00195-X 12615298

[B2] Vogel S Sardy M Glos KKHC Ruzicka T Wollenberg A The Munich outbreak of cutaneous cowpox infection: transmission by infected pet rats Acta Derm Venereol 2012 92 126 131 10.2340/00015555-1227 22041995

[B3] Cardeti G Brozzi A Eleni C Polici N D'Alterio G Carletti F Scicluna MT Castilletti C Capobianchi M Di Caro A Autorino GL Amaddeo D Cowpox virus in llama, Italy Emerg Infect Dis 2011 17 1513 1515 21801638 10.3201/eid1708.101912 PMC3381539

[B4] Megid J Borges IA Trindade GS Appolinário CM Ribeiro MG Allendorf SD Antunes JM Silva-Fernandes AT Kroon EG Vaccinia virus zoonotic infection, São Paulo State, Brazil Emerg Infect Dis 2012 18 189 191 22260819 10.3201/eid1801.110692 PMC3310104

[B5] Lewis-Jones S Zoonotic poxvirus infections in humans Curr Opin Infect Dis 2004 17 81 89 10.1097/00001432-200404000-00003 15021045

[B6] Wiser I Balicer RD Cohen D An update on smallpox vaccine candidates and their role in bioterrorism related vaccination strategies Vaccine 2007 25 976 984 10.1016/j.vaccine.2006.09.046 17074424

[B7] Bray M Pathogenesis and potential antiviral therapy of complications of smallpox vaccination Antiviral Res 2003 58 101 114 10.1016/S0166-3542(03)00008-1 12742570

[B8] Enserink M Smallpox Vaccines: Looking Beyond the Next Generation Science 2004 304 809 15131277 10.1126/science.304.5672.809a

[B9] Kenner J Cameron F Empig C Jobes DV Gurwith M LC16m8: an attenuated smallpox vaccine Vaccine 2006 24 7009 7022 10.1016/j.vaccine.2006.03.087 17052815 PMC7115618

[B10] Earl PL Americo JL Wyatt LS Eller LA Whitbeck JC Cohen GH Eisenberg RJ Hartmann CJ Jackson D Kulesh DA Martinez MJ Miller DM Mucker EM Shamblin JD Zwiers SH Huggins JW Jahrling PB Moss B Immunogenicity of a highly attenuated MVA smallpox vaccine and protection against monkeypox Nature 2004 428 182 185 10.1038/nature02331 15014500

[B11] Stittelaar KJ van Amerongen G Kondova I Kuiken T van Lavieren RF Pistoor FH Niesters HG van Doornum G van der Zeijst BA Mateo L Chaplin PJ Osterhaus ADME Modified vaccinia virus Ankara protects macaques against respiratory challenge with monkeypox virus J Virol 2005 79 7845 7851 10.1128/JVI.79.12.7845-7851.2005 15919938 PMC1143678

[B12] Wyatt LS Earl PL Eller LA Moss B Highly attenuated smallpox vaccine protects mice with and without immune deficiencies against pathogenic vaccinia virus challenge Proc Natl Acad Sci USA 2004 101 4590 4595 10.1073/pnas.0401165101 15070762 PMC384791

[B13] Belyakov IM Earl P Dzutsev A Kuznetsov VA Lemon M Wyatt LS Snyder JT Ahlers JD Franchini G Moss B Berzofsky JA Shared modes of protection against poxvirus infection by attenuated and conventional smallpox vaccine viruses Proc Natl Acad Sci USA 2003 100 9458 9463 10.1073/pnas.1233578100 12869693 PMC170940

[B14] Blanchard TJ Alcami A Andrea P Smith GL Modified vaccinia virus Ankara undergoes limited replication in human cells and lacks several immunomodulatory proteins: implications for use as a human vaccine J Gen Virol 1998 79 1159 1167 9603331 10.1099/0022-1317-79-5-1159

[B15] Ferrier-Rembert A Drillien R Tournier JN Garin D Crance JM Short- and long-term immunogenicity and protection induced by non-replicating smallpox vaccine candidates in mice and comparison with the traditional 1st generation vaccine Vaccine 2008 26 1794 1804 10.1016/j.vaccine.2007.12.059 18336966

[B16] Kennedy RB Ovsyannikova I Poland GA Smallpox vaccines for biodefense Vaccine 2009 27 D73 D79 19837292 10.1016/j.vaccine.2009.07.103 PMC2764553

[B17] Hooper JW Ferro AM Golden JW Silvera P Dudek J Alterson K Custer DM Rivers B Morris J Owens G Smith JF Kamrud KI Molecular smallpox vaccine delivered by alphavirus replicons elicits protective immunity in mice and non-human primates Vaccine 2010 28 494 511 19833247 10.1016/j.vaccine.2009.09.133 PMC2789203

[B18] Edghill-Smith Y Bray M Whitehouse CA Miller D Mucker E Manischewitz J King LR Robert-Guroff M Hryniewicz A Venzon D Meseda C Weir J Nalca A Livingston V Wells J Lewis MG Huggins J Zwiers SH Golding H Franchini G Smallpox vaccine does not protect macaques with AIDS from a lethal monkeypox virus challenge J Infect Dis 2005 191 372 381 10.1086/427265 15633096

[B19] Smith GL Vanderplasschen A Law M The formation and function of extracellular enveloped vaccinia virus J Gen Virol 2002 83 2915 2931 12466468 10.1099/0022-1317-83-12-2915

[B20] Roberts KL Smith GL Vaccinia virus morphogenesis and dissemination Trends Microbiol 2008 16 472 479 10.1016/j.tim.2008.07.009 18789694

[B21] Moss B Smallpox vaccines: targets of protective immunity Immunol Rev 2011 239 8 26 10.1111/j.1600-065X.2010.00975.x 21198662 PMC3074351

[B22] Hooper JW Custer DM Thompson E Four-gene-combination DNA vaccine protects mice against a lethal vaccinia virus challenge and elicitis appropriate antibody responses in nonhuman primates Virology 2002 306 181 195 12620810 10.1016/S0042-6822(02)00038-7 PMC9628742

[B23] Fogg C Lustig S Whitbeck JC Eisenberg RJ Cohen GH Moss B Protective immunity to vaccinia virus induced by vaccination with multiple recombinant outer membrane proteins of intracellular and extracellular virions J Virol 2004 78 10230 10237 10.1128/JVI.78.19.10230-10237.2004 15367588 PMC516428

[B24] Edghill-Smith Y Golding H Manischewitz J King LR Scott D Bray M Nalca A Hooper JW Whitehouse CA Schmitz JE Franchini G Smallpox vaccine-induced antibodies are necessary and sufficient for protection against monkeypox virus Nat Med 2005 11 740 747 10.1038/nm1261 15951823

[B25] Panchanathan V Chaudhri G Karupiah G Antiviral protection following immunization correlates with humoral but not cell-mediated immunity Immunol Cell Biol 2010 88 461 467 10.1038/icb.2009.110 20066003

[B26] Hooper JW Thompson E Wilhelmsen C Zimmerman M Ichou MA Steffen SE Schmaljohn CS Schmaljohn AL Jahrling PB Smallpox DNA vaccine protects nonhuman primates against lethal monkeypox J Virol 2004 78 4433 4443 10.1128/JVI.78.9.4433-4443.2004 15078924 PMC387704

[B27] Golden JW Josleyn MD Hooper JW Targeting the vaccinia virus L1 protein to the cell surface enhances production of neutralizing antibodies Vaccine 2008 26 3507 3515 10.1016/j.vaccine.2008.04.017 18485547

[B28] Zanotto C Pozzi E Pacchioni S Volonté L De Giuli Morghen C Radaelli A Canarypox and fowlpox viruses as recombinant vaccine vectors: a biological and immunological comparison Antiviral Res 2010 88 53 63 10.1016/j.antiviral.2010.07.005 20643163

[B29] Taylor J Weinberg R Languet B Desmettre P Paoletti E Recombinant fowlpox virus inducing protective immunity in nonavian species Vaccine 1988 6 497 503 10.1016/0264-410X(88)90100-4 2854338

[B30] Radaelli A De Giuli MC Expression of HIV-1 envelope gene by recombinant avipoxvirus Vaccine 1994 12 1101 1109 10.1016/0264-410X(94)90180-5 7998420

[B31] Parks RJ Krell PJ Derbyshire JB Nagy E Studies of fowlpox virus recombination in the generation of recombinant vaccines Virus Res 1994 32 283 297 10.1016/0168-1702(94)90078-7 8079511

[B32] Pozzi E Basavecchia V Zanotto C Pacchioni S De Giuli Morghen C Radaelli A Construction and characterization of recombinant fowlpox viruses expressing human papilloma virus E6 and E7 oncoproteins J Virol Methods 2009 158 184 189 10.1016/j.jviromet.2009.01.021 19428588

[B33] Bissa M Pacchioni S Zanotto C De Giuli Morghen C Radaelli A GFP co-expression reduces the A33R gene expression driven by a fowlpox vector in replication permissive and non-permissive cell lines J Virol Methods 2013 187 172 176 10.1016/j.jviromet.2012.09.009 23000750

[B34] Rosel JL Earl PL Weir J Moss B Conserved TAAATG Sequence at the Transcriptional and Translational Initiation Sites of Vaccinia Virus Late Genes Deduced by Structural and Functional Analysis of the Hindlll H Genome Fragment J Virol 1986 60 436 449 3021979 10.1128/jvi.60.2.436-449.1986 PMC288911

[B35] Zanotto C Pozzi E Pacchioni S Bissa M De Giuli Morghen C Radaelli A Construction and characterisation of a recombinant fowlpox virus that expresses the human papilloma virus L1 protein J Transl Med 2011 9 190 200 10.1186/1479-5876-9-190 22053827 PMC3231814

[B36] Pacchioni S Volonté L Zanotto C Pozzi E De Giuli Morghen C Radaelli A Canarypox and fowlpox viruses as recombinant vaccine vectors: an ultrastructural comparative analysis Arch Virol 2010 155 915 924 10.1007/s00705-010-0663-7 20379750

[B37] Rasband WS U.S. National Institutes of Health Bethesda, Maryland, USA http://rsb.info.nih.gov/ij/

[B38] Radaelli A De Giuli Morghen C Zanotto C Pacchioni S Bissa M Franconi R Massa S Paolini F Muller A Venuti A A prime/boost strategy by DNA/fowlpox recombinants expressing a mutant E7 protein for the immunotherapy of HPV-associated cancers Virus Res 2012 170 44 52 10.1016/j.virusres.2012.08.007 22951311

[B39] Wilton S Gordon J Dale S Identification of antigenic determinants by polyclonal and hybridoma antibodies induced during the course of infection by vaccinia virus Virology 1986 148 84 96 10.1016/0042-6822(86)90405-8 2417414

[B40] Hutin YJ Williams RJ Malfait P Pebody R Loparev VN Ropp SL Rodriguez M Knight JC Tshioko FK Khan AS Szczeniowski MV Esposito JJ Outbreak of human monkeypox, Democratic Republic of Congo, 1996 to 1997 Emerg Infect Dis 2012 7 434 438 11384521 10.3201/eid0703.010311 PMC2631782

[B41] Reed KD Melski JW Graham MB Regnery RL Sotir MJ Wegner MV Kazmierczak JJ Stratman EJ Li Y Fairley JA Swain GR Olson VA Sargent EK Kehl SC Frace MA Kline R Foldy SL Davis JP Damon IK The detection of monkeypox in humans in the Western Hemisphere New Engl J Med 2004 350 342 350 10.1056/NEJMoa032299 14736926

[B42] Schulze C Alex M Schirrmeier H Hlinak A Engelhardt A Koschinski B Beyreiss B Hoffmann M Czerny CP Generalized fatal Cowpox virus infection in a cat with transmission to a human contact case Zoonoses Public Health 2007 54 31 37 10.1111/j.1863-2378.2007.00995.x 17359444

[B43] Reynolds MG Carroll DS Olson VA Hughes C Galley J Likos A Montgomery JM Suu-Ire R Kwasi MO Jeffrey Root J Braden Z Abel J Clemmons C Regnery R Karem K Damon IK A silent enzootic of an orthopoxvirus in Ghana, West Africa: evidence for multi-species involvement in the absence of widespread human disease Am J Trop Med Hyg 2010 82 746 754 10.4269/ajtmh.2010.09-0716 20348530 PMC2844556

[B44] Rimoin AW Mulembakani PM Johnston SC Lloyd Smith JO Kisalu NK Kinkela TL Blumberg S Thomassen HA Pike BL Wolfe ND Shongo RL Graham BS Formenty P Okitolonda E Hensley LE Meyer H Wright LL Muyembe JJ Major increase in human monkeypox incidence 30 years after smallpox vaccination campaigns cease in the Democratic Republic of Congo Proc Natl Acad Sci USA 2010 107 16262 16267 10.1073/pnas.1005769107 20805472 PMC2941342

[B45] Parker S Nuara A Buller RML Schultz DA Human monkeypox: an emerging zoonotic disease Future Microbiol 2007 2 17 34 10.2217/17460913.2.1.17 17661673

[B46] Cohen J Is an old virus up to new tricks? Science 1997 277 312 313 10.1126/science.277.5324.312 9518358

[B47] Lorenzo M Galindo I Griffiths G Blasco R Intracellular localization of vaccinia virus extracellular enveloped virus envelope proteins individually expressed using a Semliki Forest virus replicon J Virol 2000 74 10535 10550 10.1128/JVI.74.22.10535-10550.2000 11044098 PMC110928

[B48] Hooper JW Golden JW Ferro AM King AD Smallpox DNA vaccine delivered by novel skin electroporation device protects mice against intranasal poxvirus challenge Vaccine 2007 25 1814 1823 10.1016/j.vaccine.2006.11.017 17240007 PMC9628994

[B49] Baxby D Paoletti E Potential use of nonreplicating vectors as recombinant vaccines Vaccine 1992 10 8 9 10.1016/0264-410X(92)90411-C 1311489

[B50] Skinner MA Laidlaw SM Eldaghayes I Kaiser P Cottingham MG Fowlpox virus as a recombinant vaccine vector for use in mammals and poultry Expert Rev Vaccines 2005 4 63 76 10.1586/14760584.4.1.63 15757474

